# Bacteria Derived from Diamondback Moth, *Plutella xylostella* (L.) (Lepidoptera: Plutellidae), Gut Regurgitant Negatively Regulate Glucose Oxidase-Mediated Anti-Defense Against Host Plant

**DOI:** 10.3390/insects15121001

**Published:** 2024-12-17

**Authors:** Qingxuan Qiao, Huiting Feng, Lu Jiao, Uroosa Zaheer, Chanqin Zheng, Li Zhou, Guifang Lin, Xiujuan Xiang, Huang Liao, Shanyu Li, Haiyan Lu, Anyuan Yin, Yussuf Mohamed Salum, Hui Wei, Wei Chen, Weiyi He, Feiying Yang

**Affiliations:** 1 State Key Laboratory for Ecological Pest Control of Fujian and Taiwan Crops, Institute of Applied Ecology, Fujian Agriculture and Forestry University, Fuzhou 350002, China; qqx177845@gmail.com (Q.Q.);; 2 International Joint Research Laboratory of Ecological Pest Control, Ministry of Education and Ministerial and Provincial Joint Innovation Centre for Safety Production of Cross-Strait Crops, Fujian Agriculture and Forestry University, Fuzhou 350002, China; 3 State Key Laboratory of Ecological Pest Control for Fujian and Taiwan Crops, Institute of Plant Protection, Fujian Academy of Agricultural Sciences, Fuzhou 350002, China; 4 Ganzhou Key Laboratory of Greenhouse Vegetable, School of Life Sciences, Gannan Normal University, Ganzhou 341000, China; 5 Institute of Biological Resources, Jiangxi Academy of Sciences, Nanchang 330029, China

**Keywords:** glucosinolate, regurgitant, bacteria, glucose oxidase, plant hormone

## Abstract

The ongoing interplay among plants, insects, and bacteria underscores the intricate balance of defense mechanisms in ecosystems. Regurgitant bacteria directly/indirectly impact plant immune responses, but the underlying mechanism is unclear. Here, we focus on the interaction between regurgitant bacteria, diamondback moth (DBM), and plant. Six culturable bacteria were isolated from DBM gut regurgitant, including three *Enterobacter* strains (RB1-3), *Micrococcus* sp. (RB4), *Staphylococcus haemolyticus* (RB5), and *Bacillus cereus* (RB6). These RB strains suppressed genes related to jasmonic acid and glucosinolate signaling pathways but had little effect on salicylic acid signaling pathway genes in *Arabidopsis thaliana* wounds. RB1 and RB5 inhibited DBM development on *A. thaliana* but not on an artificial diet. RB1 and RB5 significantly suppressed GOX genes and proteins in DBMs. However, the *Pxgox2* insect mutant strain inoculated with RB1 or RB5 did not significantly affect DBM feeding on *A. thaliana* compared to the wild type. Six RB have been functionally identified, with RB1 and RB5 negatively regulating GOX-mediated host adaptability. The deliberate addition of RB1 and RB5 can negatively affect DBM herbivory and fitness. Our study provides a molecular basis for the further application of RB for insect pest management by modulating insect–plant interactions.

## Simple Summary

In natural ecosystems, interactions among plants, insects, and bacteria create complex defense dynamics. Our study explores how specific bacteria in the gut regurgitant of diamondback moths (DBMs) suppress the plant immune response. We isolated six bacterial strains from DBM gut regurgitant and found that two of them, RB1 and RB5, effectively reduced the expression of defense-related plant genes and suppressed DBM growth on host plants. Interestingly, these bacteria also decreased the activity of DBM’s glucose oxidase, an enzyme crucial for DBM’s adaptation to plants. Our findings highlight the possibility of using DBM gut bacteria to naturally boost plant resistance, supporting sustainable pest control approaches.

## 1. Introduction

Due to their immobility, plants must adapt to biotic and abiotic environmental stresses, such as the threat posed by herbivorous insects, to ensure their survival. Plants have co-evolved with the surrounding ecological environment and have developed various defense mechanisms that combat external challenges [1,2,3,4]. In the case of plant–insect interactions, plants protect themselves against insect attacks, while insects have additionally created a set of counter-strategies in the co-evolution with plant defense systems to enable them to survive and reproduce [5]. These counter-strategies mainly occur through effectors release [6], which decreases the adaptive cost when feeding on plants by influencing plant nutrition, interfering with plant defense signals, and lowering the toxicity of plant defense chemicals [7,8,9]. In Lepidoptera, effectors are primarily identified in larval saliva and regurgitant. The saliva is mainly secreted by the labial glands and released through the spinneret [10], while the regurgitant comes from the gut of the digestive system [11,12]. During the process of insects feeding on host plants, they inhibit the plants’ defense response by releasing specific effectors contained in saliva or regurgitant.

Currently, many effectors have been identified in different insects. In Hemiptera, candidate effector 6 (Al6) of the mirid bug, *Apolygus lucorum*, can encode an active glutathione peroxidase, inhibiting the accumulation of reactive oxygen species induced by the two pathogen-associated molecular patterns of infiltration-released elicitin 1 (INF1) and flagellin-derived peptide 22 (Flg22), thereby inhibiting pattern-triggered immune-induced cell death [13]. The salivary effector protein, an immunosuppressive agent, of whitefly, *Bemisia tabaci*, directly interacts with the immune regulator WRKY transcription factor 33 in plants, inhibiting the activation of immune-related genes and thus inhibiting plant immune signaling [14]. In addition, a venom-like protein, HARP1, has been isolated from the oral secretions (OS) of cotton bollworm, *Helicoverpa armigera*, interacting with multiple JASMONATE-ZIM-domain (JAZ) proteins of *A. thaliana* and cotton, *Gossypium hirsutum*, preventing CORONATINE INSENSITIVE1(COI1)-mediated JAZ degradation and thus blocking JA signaling output [9]. The glucose oxidase (GOX), functioning in inhibiting plants’ defense responses [7,15,16], is an effector common in Lepidoptera, initially discovered in the saliva of the caterpillar, *Helicoverpa zea* (*H. zea*) [17]. As one of the most characteristic salivary enzymes in Lepidoptera larvae, GOX is generally believed to be lacking in gut regurgitant. Nevertheless, in the diamondback moth (DBM), *Plutella xylostella* (L.) (Lepidoptera: Plutellidae), the sole identified effector is GOX, originating from the gut regurgitant, which suppresses host plant defenses by generating H_2_O_2_, which can control the antagonistic phytohormone signaling pathways of salicylic acid (SA) and jasmonic acid (JA) [18].

When insects feed on host plants with chewing mouthparts, they leave regurgitant on the wound site, considered the primary source of ruminant insect effectors [5]. In addition to effectors, recent studies have also shown that regurgitant contain various microbial communities that may impact plant–insect interactions [11,19,20]. Plants’ ability to perceive clues related to herbivores present in OS suggests that this may be related to exposure to regurgitant bacteria (RB) and effectors [1,21,22]. Many studies have demonstrated RB are essential in mediating the interaction between insects and plants [19,23]. This regulation can be achieved by directly exploiting the antagonistic relationship between the plant JA and SA signaling pathways [24]. For example, the colorado potato beetle, *Leptinotarsa decemlineata*, secretes bacteria that can activate the SA signaling pathway in plants and inhibit the JA signaling pathway, and decrease the plant defense responses to insects [10]. In another system with a closely related species, the false potato beetle, *Leptinotarsa juncta*, secreted bacteria are regarded as microbial threats by tomato plants, thereby activating the SA signaling pathway to antagonize the JA signaling pathway [11]. However, RB do not always benefit insects feeding on host plants. It has been discovered that after *H. zea* larvae were inoculated with their own *Enterobacter ludwigii* (*E. ludwigii*) from OS, the JA signaling pathway genes in the host plant were indirectly induced to express during *H. zea* feeding, enhancing the plants’ defense against insects and hindering their feeding activities [25]. In conclusion, gut RB play a broader and complex role in regulating insect–plant interactions.

The DBM is a worldwide pest, mainly feeding on cruciferous plants. Defensive and counter-defensive strategies are present in all aspects of adapting DBMs to the host plant during long-term co-evolution [26]. Effectors from insect OS play a significant role in weakening plant defense responses. However, the current effector studies in DBMs are limited to GOX. Our previous research identified the GOX effector in the gut regurgitant of DBMs, which functions as an effector and catalyzes the conversion of glucose in the host plant into H_2_O_2_. The increased environmental H_2_O_2_ levels can effectively disrupt the wound-induced JA-mediated defense response of the host plant against insect herbivores with chewing mouthparts by promoting the SA signaling pathway, which antagonizes the JA signaling pathway [18]. Furthermore, in addition to effectors, a substantial amount of RB is released during the feeding process of DBMs, but whether RB directly regulate the plant defense response or indirectly regulate the effector GOX-mediated plant defense response is currently unclear. The composition and function of the insect gut microbiota are generally understood [27], and the preparation techniques for gut bacteria-free insects are relatively advanced [28,29].

In this study, we employed the gut RB of DBMs and the interaction between DBMs and cruciferous plants as our research models. To investigate the direct or indirect molecular mechanisms underlying the gut RB regulation of insect–plant interactions, we chose insects fed on an artificial diet to minimize the disturbance of the bacteria carried by feeding on plants. Here, we isolated and identified different culturable bacteria from the regurgitant of DBMs using traditional microbial isolation and pure culture methods, and verified their function in DBM feeding on plants. Furthermore, we employed previously obtained *Pxgox2* mutants through Clustered Regularly Interspaced Short Palindromic Repeat/CRISPR-associated protein 9 (CRISPR/Cas9) to explore the molecular mechanisms of the gut RB interaction with the GOX effector of DBMs.

## 2. Materials and Methods

### 2.1. Insect Strains

The larvae of a wild-type (WT) DBM strain were collected from vegetable fields of cruciferous crops in the suburban area of Fuzhou City, Fujian Province, China (26.08 N, 119.28 E) on April, 2004 and were continuously reared at a constant temperature of 23 ± 1 °C, a relative humidity of 65 ± 5%, and under controlled conditions, with a photoperiod of 16:8 h (light/dark) in a culture room. The wild-type DBMs were reared to adults in small mesh cages (500 mm × 500 mm × 500 mm) containing fresh radish seedlings. Adults were given a 10% honey solution to supplement their nutrition and promote oviposition. Upon hatching, the larvae were further reared on radish leaves. Our laboratory has proved that the growth and development of DBMs reared on radish cotyledon are normal and healthy [30]. The artificial diet (AD) DBM strain was introduced from the Institute of Zoology Chinese Academy of Science (Beijing, China) and was reared in a culture room. Larvae were fed with an artificial diet in transparent plastic containers (150 mm × 100 mm × 50 mm) consisting of 1.8 g agar and 6 g yeast powder, boiled and cooled in 75 mL pure water, supplemented with 11.25 g wheat germ meal, 0.3 g vitamin powder (containing vitamin D3, vitamin B1, vitamin B2, vitamin B6, folic acid, niacin, and vitamin C), 0.3 g sorbic acid, 0.3 g ascorbic acid, 0.3 g ethyl 4-hydroxybenzoate, 3 g sucrose, 0.9 g powdered radish seeds (Nanpan Prefecture), 0.3 mL rapeseed oil, and 0.03 mL linoleic acid [31]. The *Pxgox2* mutant strain of DBM was created using CRISPR/Cas9 in our laboratory [18]. The mutants of the DBM were used for the assay of host transfer after being continuously reared for four generations on an artificial diet without the addition of radish seed powder to eliminate the potential interference from plant defense chemicals.

Bacteria-free larvae were obtained by using newly laid AD eggs, which were subjected to sequential treatments of sterile water (3 min), 1.5% sodium hypochlorite solution (30 s), 75% ethyl alcohol (*v*/*v*) (3 min), and sterile water (3 min) three times. The treated eggs were then dried on sterilized filter paper towels in a laminar flow cabinet. The hatched larvae were fed with a sterilized artificial diet without antibiotic, with all the materials used subjected to sterilization or disinfection procedures, and reared in a bacteria-free environment until the different age stages required for the experiment.

### 2.2. Host Plants

The radish was the Nanpan Prefecture white radish line, and the wild-type *A. thaliana* (Col-0 ecotype) was purchased from Kaiyi Biotechnology (Shanghai, China). The plants were grown in rectangular plastic trays (450 mm × 300 mm × 70 mm) filled with nutrient-rich soil (peat soil/vermiculite = 1:1) and placed in a culture room under controlled conditions, with a photoperiod of 16:8 h (light/dark), a light intensity of 2000 lux, a constant temperature of 23 ± 1 °C, and the relative humidity maintained at 65 ± 5%. The radish seedlings were utilized for feeding insects when the cotyledons were fully expanded (approximately 1 week old), and the one-month-old *A. thaliana* leaves were further used for feeding assays.

### 2.3. Defense Responses of the Host Plants to Different Treatments

Because the larvae of DBMs are too small to collect enough gut regurgitant for the further experiment, we treated the *A. thaliana* leaves with insect gut grinding liquid prepared from the dissected guts of 50 4th instar larvae. The guts were dissected under sterile conditions in phosphate-buffered saline (PBS, pH 7.4; Solarbio, Beijing, China) and homogenized in 500 µL of PBS to produce the gut homogenate. In brief, a volume of 20 µL of the gut content of the 4th instars was applied on the physical wound (5 cm^2^) of the one-month-old *A. thaliana* leaves. The gut grinding liquid was used in three forms: untreated, pretreated with proteinase K (Solarbio, China) at 37 °C for 30 min to specifically degrade effector proteins, or boiled at 95 °C for 10 min to eliminate both bacteria and effector proteins. The unwounded leaves and the leaves treated with PBS were set as the controls. The time point of 8 h after treatment (hat) was selected based on evidence from previous studies, which demonstrated significant transcriptional changes in JA, SA, and GS pathways between 1 and 24 h after herbivore feeding [32]. We then measured the gene expression of defense-related phytohormone pathways, JA, SA, and GSs metabolism at 8 h after treatment (hat), when the associated genes were extensively influenced by the larval feeding of DBMs.

### 2.4. Isolation of Culturable Bacteria from Larval Gut Regurgitant

The newly emerged 4th instars were used to isolate culturable bacteria from gut regurgitant. The larvae were surface-sterilized by 75% ethyl alcohol (*v*/*v*) for 1 min and rinsed with sterile water for 1 min three times. Due to the small amount of gut regurgitant, each replicate for the isolation of culturable bacteria consisted of approximately 150 individuals by touching the larval abdomen gently. The mixture of gut regurgitant was diluted in 5 gradient concentrations. Twenty µL of the final collection was individually used to coat the culture solid media of Luria–Bertani (LB), Nutrient Agar (NA), Salmonella–Shigella (SS), and Enterococcus Agar (EA).

The final rinse of water for the body surface was set as a control to eliminate surface microbes. A Petri dish containing sterile water exposed to the air in a clean bench was set as an environmental control to exclude air-borne microbes. A volume of 90 µL of each control water was coated on the LB plate, with three duplicates for the treatment and control groups. The plates were cultured at a constant 37 °C in an incubator. A single colony of the culturable bacteria was isolated, based on the phenotypic difference, and purified through continuous culture for five generations on LB.

### 2.5. Sequence Alignment and Phylogenetic Analysis

The DNA of the bacterial monoclonal strains from the gut regurgitant of DBMs was extracted using a bacterial genomic DNA extraction kit (Qiagen, Hilden, Germany). Taking the genomic DNA as a template, the bacterial 16S rDNA universal primers 27F and 1492R were used for PCR amplification (Table S1). A standard PCR program was applied, with 95 °C for 3 min and 34 cycles at 95 °C for 30 s, 55 °C for 30 s, and 72 °C for 1 min, and a final 15 min extension at 72 °C. The amplified DNA products were sent to Biosune Biological Biotechnology Company (Shanghai, China) for both strands sequencing. The 16S rRNA sequences of the six culturable RB were submitted to NCBI GenBank (accession numbers: MT321481, MT321505, MT321506, MT321507, MT321508, and MT321509, corresponding to the strains of RB1, RB2, RB3, RB4, RB5, and RB6, respectively). The alignment and phylogenetic analysis of the 16S rRNA gene sequences were performed using the built-in MUSCLE program and neighbor-joining (NJ) method of Molecular Evolutionary Genetics Analysis (MEGA) X software (version, 10.0.5), respectively, with the default parameters.

### 2.6. RNA Extraction, cDNA Synthesis, and qRT-PCR

RNA extraction was performed according to the instructions of the RNA kit (OMEGA, Bio-Tek, Norcross, GA, USA), following the manufacturer’s protocol, and quantified by Nanodrop 2000 (ThermoFisher Scientific, Waltham, MA, USA). cDNA was synthesized using a reverse transcription kit (QIAGEN, Hilden, Germany), following the manufacturer’s protocol. The expression levels of *Pxgoxs* in DBMs were measured to investigate the interaction between gut RB and *Pxgoxs.* The *P. xylostella rpl32* gene (Genome Database ID: Px008022) was used as the internal control in the qRT-PCR analysis. Additionally, the expression levels of defense-related genes in *A. thaliana* after feeding by DBM larvae at 2 h, 4 h, and 8 h were selected as the time points for detecting the effects of gut RB on the gene regulation of the signaling pathways of JA, SA, and GSs. The *AtActin2* (At3g18780) was used as the internal reference for normalization during qRT-PCR analysis. Primer sequences for these genes are listed in Table S1. A standard qRT-PCR program was performed in the ABI QuantStudioTM 6 Flex Real-time PCR system (Life Technologies, Thermo Fisher Scientific, Carlsbad, CA, USA), with 95 °C for 3 min and 40 cycles at 95 °C for 15 s, 60 °C for 15 s, and 72 °C for 30 s. The qRT-PCR data were analyzed using the 2−ΔCt method.

### 2.7. Protein Extraction and Western Blot (WB)

The radioimmunoprecipitation assay (RIPA) cracking method was used to extract protein [33]. Frozen samples of DBM 3rd instar larvae treated by different bacterial strains were thawed on the ice, and RIPA lysate (Solarbio, Beijing, China), protease inhibitor (phenylmethylsulfonyl fluoride, Solarbio, Beijing, China), and protease stabilizer (BestBio, Shanghai, China) were added immediately. After vortex mixing, the samples were decomposed at 4 °C for 30 min, during which the samples were mixed with oscillations. Subsequently, the samples were centrifuged at 4 °C at 14,000× *g* for 10 min, and the total protein was recovered from the supernatant in a pre-cooled clean centrifuge tube. The protein concentration was determined using an Omni-Easy™ ready-to-use BCA protein quantification kit (Tagene, Guangzhou, China). A sodium dodecyl sulfate polyacrylamide gel electrophoresis (SDS-PAGE) gel with a 12.5% concentration was prepared using the Omni-Easy™ One-Step PAGE Rapid Preparation Kit (Tagene, Guangzhou, China), and the boiling denatured proteins (20 µg/lane) were then subjected to electrophoresis. Next, the proteins were transferred onto a PVDF membrane (Millipore, Darmstadt, Germany) using a DYCP-40E electrophoretic membrane transfer apparatus (Bio-rad, Hercules, CA, USA). After blocking the membranes with 3% skimmed milk in tris-buffered saline containing 0.5% Tween 20 (Solarbio, Beijing, China), the immunoreaction was performed using the primary antibody for PxGOX2 (1:1000) overnight at 4 °C. The membranes were then washed with 1 × tris-buffered saline with tween (TBST), and a second antibody of goat anti-rabbit IgG (1:5000) was conjugated with horseradish peroxidase (Affinity Biosciences, Cincinnati, OH, USA) for 2 h at room temperature. According to the manufacturer’s protocol, a color reaction was conducted using enhanced chemiluminescence (ECL) Western blotting reagent (Bio-Rad, Hercules, CA, USA), and image collection was carried out by the Fusion FX5 imaging system (Vilber Lourmat, Marne-la-Vallée, France). Tubulin was used as the loading control, and its corresponding antibody (1:1000, Abcam, Cambridge, UK) was included in parallel. The imaging results were converted with ImageJ software (version 1.53a) for subsequent data analysis.

### 2.8. Effects of Gut RB on Plant Defense Responses

Six different bacterial strains were individually inoculated into LB liquid medium, cultured under 37 °C, and shaken overnight, and the bacteria concentration was adjusted to OD600 = 1.0. A volume of 20 µL bacterial solution was applied on the physical wound (5 cm^2^) of the one-month-old *A. thaliana* leaves for 2 h, 4 h and 8 h. A sterile liquid LB solution treatment was used as a control. The expression levels of defense-related genes in *A. thaliana* leaves in JA, SA, and GSs metabolism signaling pathways were also detected. Six leaves were used as one replicate; each treatment contained three biological replicates.

### 2.9. Fitness Comparison After Inoculation of Gut RB

Six bacterial isolates were grown in LB media overnight for larval gut inoculation and fitness comparison in DBMs. Each bacterium was collected by centrifuging and resuspended in sterile water to an OD600 value of 1.0. An artificial diet (0.5 g) mixed with each bacterial suspension (200 μL) was used to feed the newly emerged bacteria-free 3rd instars. Fitness parameters (larval weight at the 4th instar, developmental duration of larvae, larval mortality, pupal weight, developmental duration of pupa, and eclosion rate) of the treated individuals were compared. Bacteria-free larvae were acquired by sterilized eggs and an artificial diet and rearing environment [25]. Bacteria-free larvae fed with the six different bacterial diets for 24 h were transferred to *A. thaliana* leaves to investigate the same fitness parameters. Bacteria-free larvae fed with a sterile diet were set as a control. In addition, the 3rd instar bacteria-free *Pxgox2* mutant larvae fed with RB1 and RB5 bacterial diets for 24 h were transferred to *A. thaliana* leaves to study the same fitness parameters. Mutant larvae fed with a sterile diet were set as the control. The diets or leaves were replaced every 48 h. Each replicate consisted of thirty larvae, and three replicates were conducted for each treatment.

### 2.10. Statistical Analysis

GraphPad Prism software (version 9.0.0, Inc., San Diego, CA, USA) was used for plotting. The data of relative gene expression values and biological parameters were statistically analyzed using SPSS software (version 21.0, Armonk, NY, USA). Data are shown as the mean ± standard error (SE). Multiple datasets were compared using one-way ANOVA, followed by Tukey’s post hoc test to determine the difference in corresponding parameters between treatments or controls, which was taken to be significant at a *p*-value < 0.05.

## 3. Results

### 3.1. Suppression of Gut Homogenate Containing RB of DBM on the Defense Responses of Host Plant

**Figure 1:**
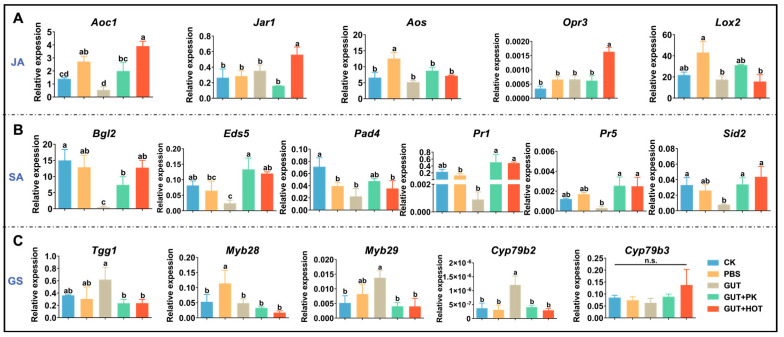
Suppression of gut homogenate containing regurgitant bacteria of diamondback moth on the defense responses of host plant. (**A**) Expression of genes associated with JA biosynthesis and signaling. (**B**) SA signaling and response. (**C**) Expression of genes involved in the biosynthesis and metabolism of glucosinolates. *Aoc1*, allene oxide cyclase 1; *Jar1*, JA-amino acid synthetase; *Aos*, allene oxide synthase; *Opr3*, 12-oxophytodienoic acid (OPDA) reductase 3; *Lox2*, lipoxygenase; *Bgl2*, β-1,3-glucanase 2. *Eds5*, enhanced disease susceptibility 5; *Pad4*: phytoalexin-deficient 4; *Pr1*, pathogenesis-related protein 1; *Pr5*, pathogenesis-related protein 5; *Sid2*, salicylic acid-induction deficient 2; *Tgg1*, β-thioglucoside hydrolase; *Myb28*, MYB DNA-binding domain transcription factor 28; *Myb29*, MYB DNA-binding domain transcription factor 29; *Cyp79b2*, cytochrome P450 monooxygenase 79b2; *Cyp79b3*, cytochrome P450 monooxygenase 79b3. CK, physically wounded leaves; PBS, CK plus PBS buffer; GUT, PBS plus gut grinding liquid; GUT+PK, PBS plus gut grinding liquid pretreated with proteinase K; GUT+HOT, PBS plus gut grinding liquid pretreated in hot water. Each column in the figure represents the mean standard error (SE) based on three replicates (*n* = 3). A significant difference analysis for the different treatments was performed using one-way ANOVA followed by Tukey’s post hoc test. Different letters indicate significant difference at *p*-value < 0.05. n.s., not significant. The sequences of all primers used in this experiment, including standard primers, are listed in Table S1 of the Supplementary Materials.

To investigate whether the RB of the DBM affect the defense responses of host plants, we performed a physical injury on *A. thaliana* leaves and treated them with insect gut homogenate for 8 h. Subsequently, we measured the gene expression of plant defense-related hormone pathways, SA, JA, and GSs. The results showed a significant suppression of genes related to the SA signaling pathway (Figure 1B), partial suppression of *AOC1* and *LOX2* in the JA signaling pathway (Figure 1A), and no consistent changes in GSs metabolism gene expression (Figure 1C). Furthermore, we added protease K separately to the gut homogenate to eliminate the effect of effector proteins. The results showed that the inhibitory effect on gene expression in the JA and SA signaling pathways was partially eliminated. Finally, heat treatment was used to eliminate the influence of effector proteins and gut microbes, resulting in the elimination of the inhibitory effect on gene expression in the JA and SA signaling pathways (Figure 1).

### 3.2. Isolation and Identification of Culturable Gut RB from DBM

**Figure 2:**
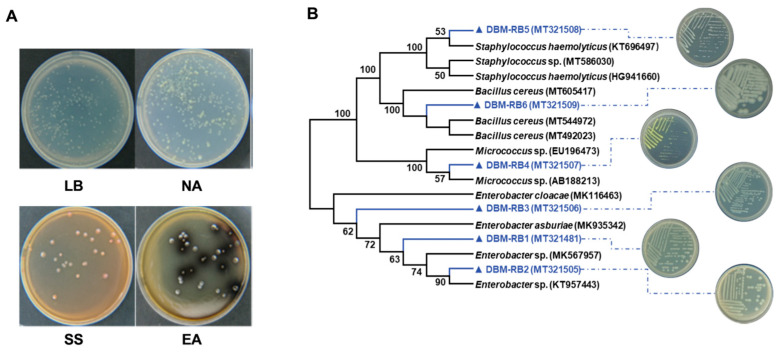
Isolation and identification of culturable gut regurgitant bacteria (RB) from the diamondback moth (DBM). (**A**) Isolation of bacteria from larval gut regurgitant in the culture media of Luria–Bertani (LB), Nutrient Agar (NA), Salmonella–Shigella (SS), and *Enterococcus* Agar (EA). (**B**) A phylogenetic tree was constructed based on the full-length 16S rRNA sequences and phenotypic characteristics of six bacteria isolated from the larval regurgitant of DBMs. Each bacterium was identified with its corresponding accession number from NCBI, highlighted in blue and represented by a triangle on the tree.

To isolate individual culturable bacteria from regurgitant, we utilized four different solid culture media, including LB, NA, SS, and EA, to culture collected gut regurgitant sample.s RB abundance was higher in LB and NA media, with SS producing light red colonies and EA producing black colonies (Figure 2A). Subsequently, pure cultures of RB were isolated on LB solid media to obtain single colony strains. The strains belonging to the three major bacterial phyla were identified based on 16S rRNA gene sequencing alignment (Supplementary Figure S1) and phylogenetic tree analysis (Figure 2B). The Proteobacteria are predominantly composed of three species: *Enterobacter* sp1. (RB1), *Enterobacter* sp2. (RB2), and *Enterobacter asburiae* (RB3). The Firmicutes consist of *Staphylococcus haemolyticus* (RB4) and *Bacillus cereus* (RB5), while Actinobacteria are represented by *Micrococcus* sp. (RB6) (Figure 2B and Table S3).

### 3.3. Gut RB from DBM Suppress Defense-Related Gene Expression of Host Plant

**Figure 3:**
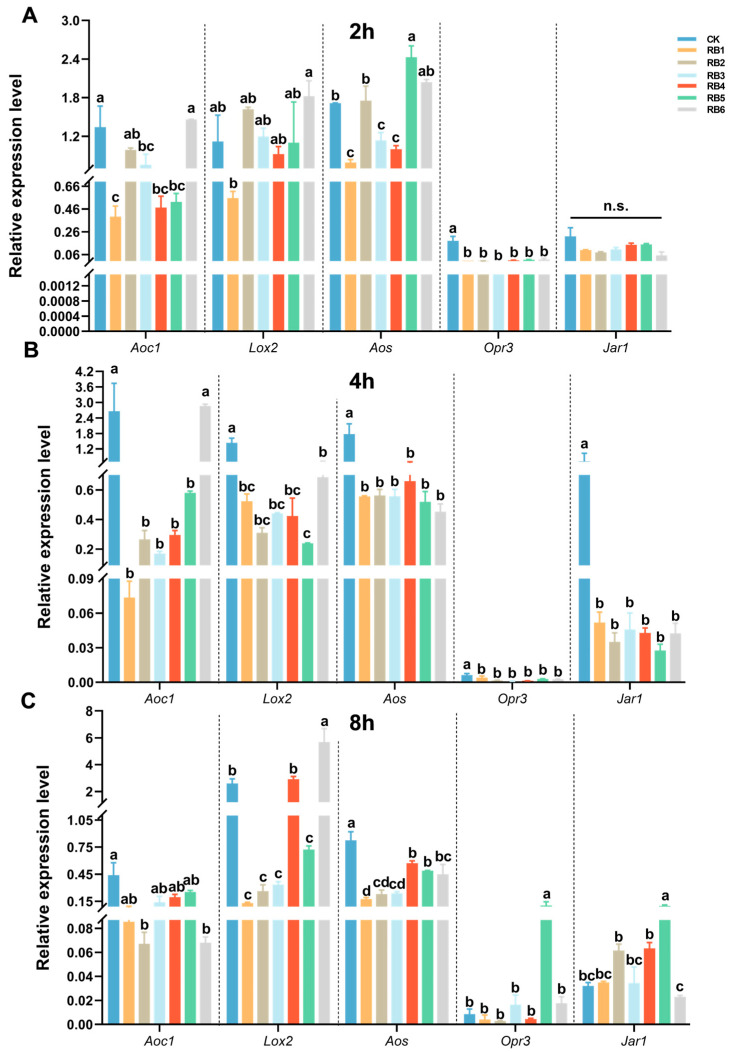
Expression profiling of genes involved in jasmonic acid (JA) biosynthesis and signaling in *Arabidopsis* leaves after treatment with larval gut regurgitant bacteria (RB) of diamondback moth. Gene expression analysis at (**A**) 2 h, (**B**) 4 h, and (**C**) 8 h after treatment. *Aoc1*, allene oxide cyclase 1; *Lox2*, lipoxygenase 2; *Aos*, allene oxide synthase; *Opr3*, 12-oxophytodienoic acid (OPDA) reductase 3; *Jar1*, JA-amino acid synthetase. CK, physically wounded leaves treated with LB media; RB, regurgitant bacteria. Each number of RB represents the larvae inoculated with the corresponding regurgitant bacterium. Each column in the figure represents the mean ± SE (*n* = 3). A significant difference analysis for the different treatments was performed using one-way ANOVA followed by Tukey’s post hoc test. Different letters indicate significant difference at *p*-value < 0.05. n.s., not significant.

To investigate how gut RB affect the adaptation of DBM to host plants, we treated *A. thaliana* leaves with liquid cultures of the six RB. We selected 2 h, 4 h, and 8 h as time points to measure the expression levels of genes related to the JA, SA, and GSs signaling pathways affected by RB. We found that 2 h after treatment (hat) with bacterial culture, the expression levels of *Aoc1* (*F* = 11.636, *df* = 6, 20, *p* < 0.001) and *Opr3* (*F* = 32.190, *df* = 6, 20, *p* < 0.001) in the JA signaling pathway were significantly downregulated in all six treatments. After 4 hat, the expression levels of *Lox2* (*F* = 22.592, *df* = 6, 20, *p* < 0.001), *Aos* (*F* = 7.639, *df* = 6, 20, *p =* 0.002), *Opr3* (*F* = 32.190, *df* = 6, 20, *p* < 0.001), and *Jar1* (*F* = 4.238, *df* = 6, 20, *p =* 0.002) in the JA signaling pathway were significantly downregulated in all six treatments. After 8 hat, the expression levels of *Aoc1* (*F* = 3.613, *df* = 6, 20, *p* < 0.031) and *Aos* (*F* = 16.234, *df* = 6, 20, *p* < 0.001) in the JA signaling pathway were significantly downregulated in all six treatments (Figure 3).

**Figure 4:**
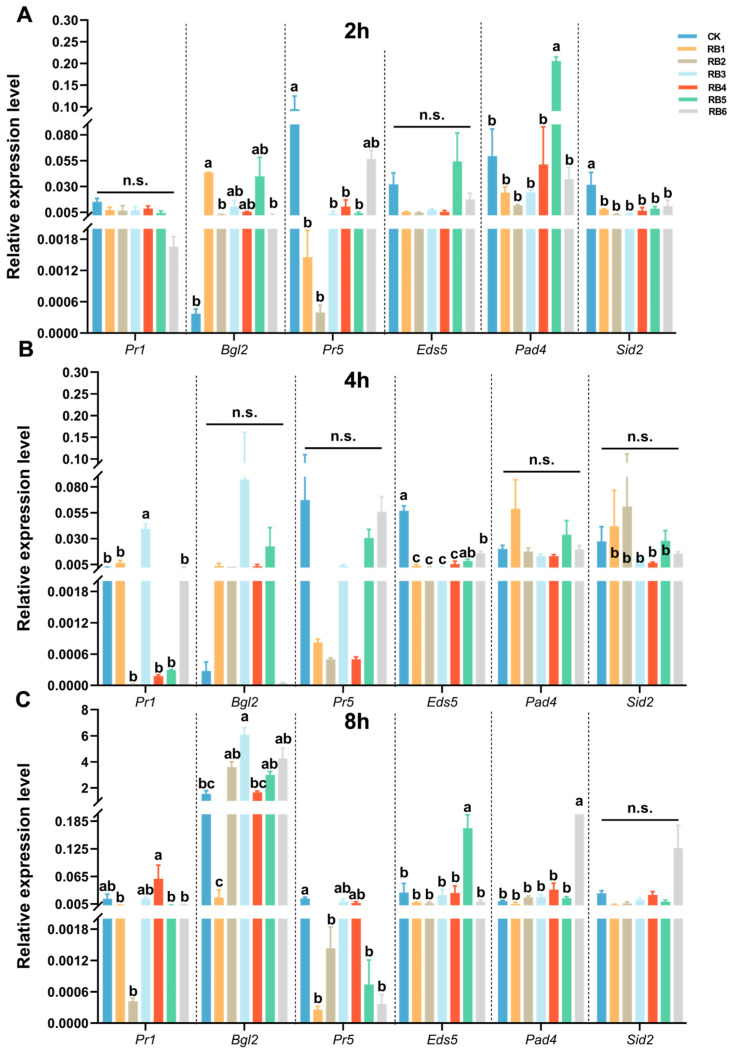
Expression profiling of genes involved in salicylic acid (SA) signaling and response in *Arabidopsis* leaves after treatment with larval gut regurgitant bacteria (RB) of diamondback moth. Gene expression analysis at (**A**) 2 h, (**B**) 4 h, and (**C**) 8 h after treatment. *Pr1*, pathogenesis-related protein 1; *Bgl2*, β-1,3-glucanase 2; *Pr5*, pathogenesis-related protein 5; *Eds5*, enhanced disease susceptibility 5; *Pad4*: phytoalexin-deficient 4; *Sid2*, salicylic acid-induction deficient 2. CK, physically wounded leaves treated with LB media; RB, regurgitant bacteria. Each number of RB represents the larvae inoculated with the corresponding regurgitant bacterium. Each column in the figure represents the mean ± SE (*n* = 3). A significant difference analysis for the different treatments was performed using one-way ANOVA followed by Tukey’s post hoc test, Different letters indicate significant difference at *p*-value < 0.05. n.s., not significant.

Similarly, after 2 hat with bacterial culture, the expression levels of *Pr5* (*F* = 7.308, *df* = 6, 20, *p* = 0.020) and *Sid2* (*F* = 3.720, *df* = 6, 20, *p* = 0.020) in the SA signaling pathway were significantly downregulated in all six treatments. After 4 hat, the expression levels of *Eds5* (*F* = 76.408, *df* = 6, 20, *p* < 0.001) in the SA signaling pathway were significantly downregulated in all six treatments. After 8 hat, the expression levels of *Pr5* (*F* = 20.392, *df* = 6, 20, *p* < 0.001) in the SA signaling pathway were significantly downregulated in all six treatments (Figure 4).

**Figure 5:**
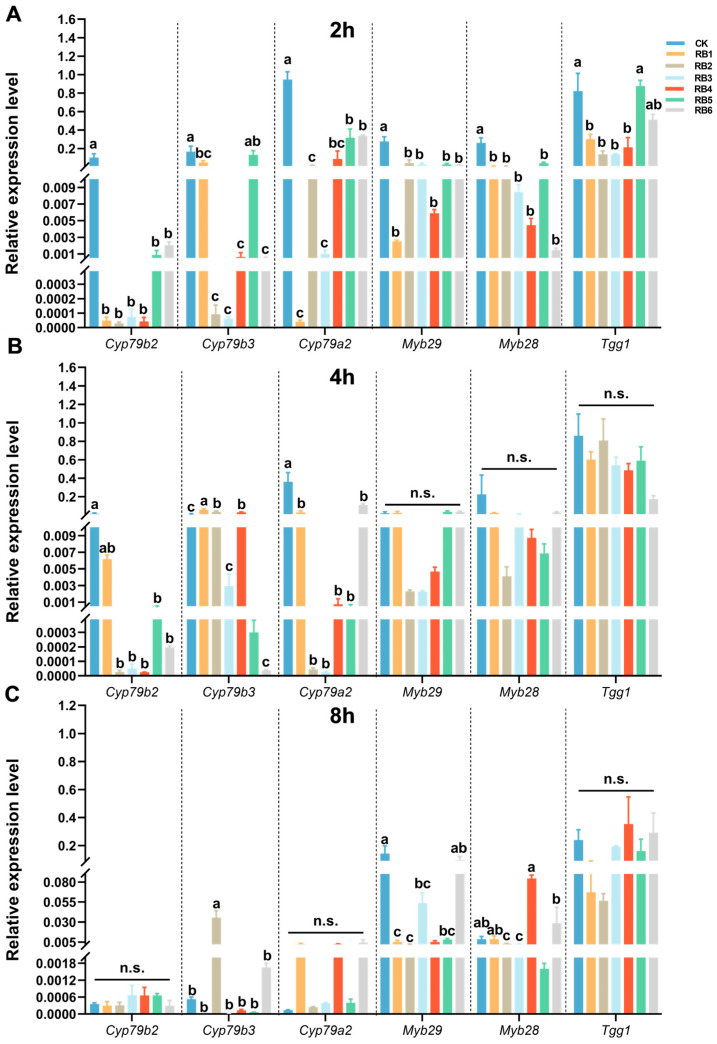
Expression profiling of genes related to glucosinolate biosynthesis and metabolism in *Arabidopsis* leaves after treatment with larval gut regurgitant bacteria (RB) of diamondback moth. Gene expression analysis at (**A**) 2 h, (**B**) 4 h, and (**C**) 8 h after treatment. *Cyp79b2*, cytochrome P450 monooxygenase 79b2; *Cyp79b3*, cytochrome P450 monooxygenase 79b3; *Cyp79a2*, cytochrome P450 monooxygenase 79a2; *Myb29*, MYB DNA-binding domain transcription factor 29; *Myb28*, MYB DNA-binding domain transcription factor 28; *Tgg1*, β-thioglucoside hydrolase. CK, physically wounded leaves treated with LB media; RB, regurgitant bacteria. Each number of RB represents the larvae inoculated with the corresponding regurgitant bacterium. Each column in the figure represents the mean ± SE (*n* = 3). A significant difference analysis for the different treatments was performed using one-way ANOVA followed by Tukey’s post hoc test. Different letters indicate significant difference at *p*-value < 0.05. n.s., not significant.

In addition, after 2 hat with bacterial culture, the expression levels of *Cyp79b2* (*F* = 6.461, *df* = 6, 20, *p* = 0.002), *Cyp79b3* (*F* = 10.776, *df* = 6, 20, *p* < 0.001), *Cyp79a2* (*F* = 30.833, *df* = 6, 20, *p* < 0.001), *Myb29* (*F* = 16.181, *df* = 6, 20, *p* < 0.001), and *Myb28* (*F* = 19.419, *df* = 6, 20, *p* < 0.001) in the GSs signaling pathway were significantly downregulated in all six treatments. After 4 hat, the expression levels of *Cyp79b2* (*F* = 4.169, *df* = 6, 20, *p* = 0.013) and *Cyp79a2* (*F* = 11.972, *df* = 6, 20, *p* < 0.001) in the GSs signaling pathway were significantly downregulated in all six treatments. After 8 hat, the expression levels of *Myb29* (*F* = 8.622, *df* = 6, 20, *p* < 0.001) in the GSs signaling pathway were significantly downregulated in all six treatments (Figure 5).

### 3.4. RB1 and RB5 Effectively Suppress DBM Performance on Host Plants

**Figure 6:**
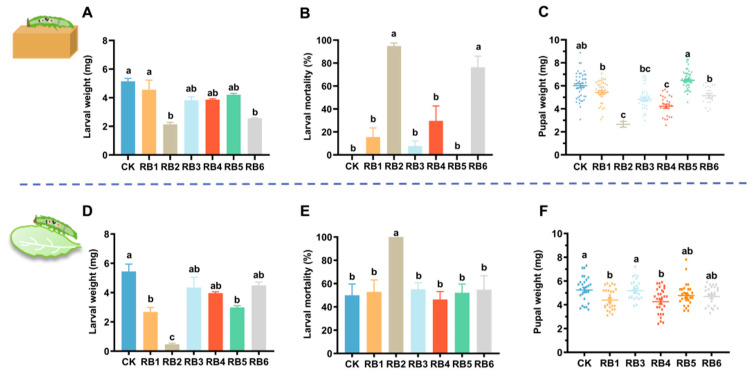
Impact of gut regurgitant bacteria (RB) on the growth and development of diamondback moth (DBM) larvae fed on an artificial diet and *A. thaliana*. The larval weight at the 4th instar (**A**,**D**), larval mortality (**B**,**E**), and pupal weight (**C**,**F**) were compared for the larvae reared on an artificial diet and the host plant *Arabidopsis*, each of which has been inoculated with a different single strain of the bacteria isolated from the larval regurgitant of DBM. Each column represents the mean ± standard error (*n* = 3). A significant difference analysis for the different treatments was performed using one-way ANOVA followed by Tukey’s post hoc test. Different letters indicate significant difference at *p*-value < 0.05. CK, larvae with sterile gut without inoculation of bacteria; RB, regurgitant bacteria. Each number of RB represents the larvae inoculated with the corresponding regurgitant bacterium. Due to the high mortality at the larval stage treated by RB2, the biological parameters of the subsequent developmental stages of this treatment were absent.

To study the impact of gut RB on the adaptability of DBMs feeding on host plants, we evaluated the biological parameters of bacteria-free DBM larvae fed on an artificial diet or *A. thaliana* after inoculation with six different bacterial strains. The results of DBM larvae fed on an artificial diet after treatment with bacterial culture showed that the larvae’s weight was significantly lower in the RB2 and RB6 treatments compared to the control group (*F* = 13.559, *df* = 6, 20, *p* < 0.001) (Figure 6A). RB2 treatment exhibited a significant larval mortality rate of 94.9% in DBM larvae, which was significantly higher than the control group (*F* = 29.127, *df* = 6, 20, *p* < 0.001) (Figure 6B). The pupal weight of DBMs under RB2, RB4, and RB6 treatments was significantly lower than the control group (*F* = 21.875, *df* = 6, 206, *p* < 0.001) (Figure 6C). Compared to the control group, there were no significant changes in the larval developmental period and pupal eclosion rate of DBMs treated with bacterial culture (Supplementary Figure S2). Furthermore, the results of DBMs feeding on *A. thaliana* after treatment with bacterial culture showed that the larval weight was significantly lower in RB1, RB2, and RB5 treatments compared to the control group (*F* = 20.051, *df* = 6, 20, *p* < 0.001) (Figure 6D). The mortality rate of DBM larvae under RB2 treatment (100%) was significantly higher than the control group (50%) (*F* = 4.998, *df* = 6, 20, *p* < 0.001) (Figure 6E). The pupal weight of DBMs under RB1 and RB4 treatments was significantly lower than the control group (*F* = 4.743, *df* = 5, 156, *p* < 0.001) (Figure 6F). These results indicate that RB1, RB2, RB4, and RB5 have adverse effects on the growth and development of DBMs feeding on *A. thaliana*, while RB2, RB4, and RB6 have adverse effects on the growth and development of DBMs feeding on artificial feed. Therefore, RB1 and RB5 were selected for further insect–bacteria interaction bioassays to exclude bacterial strains that negatively affected the performance of DBMs on artificial feed.

### 3.5. RB1 and RB5 Negatively Regulate GOX-Mediated DBM Host Adaptability

**Figure 7:**
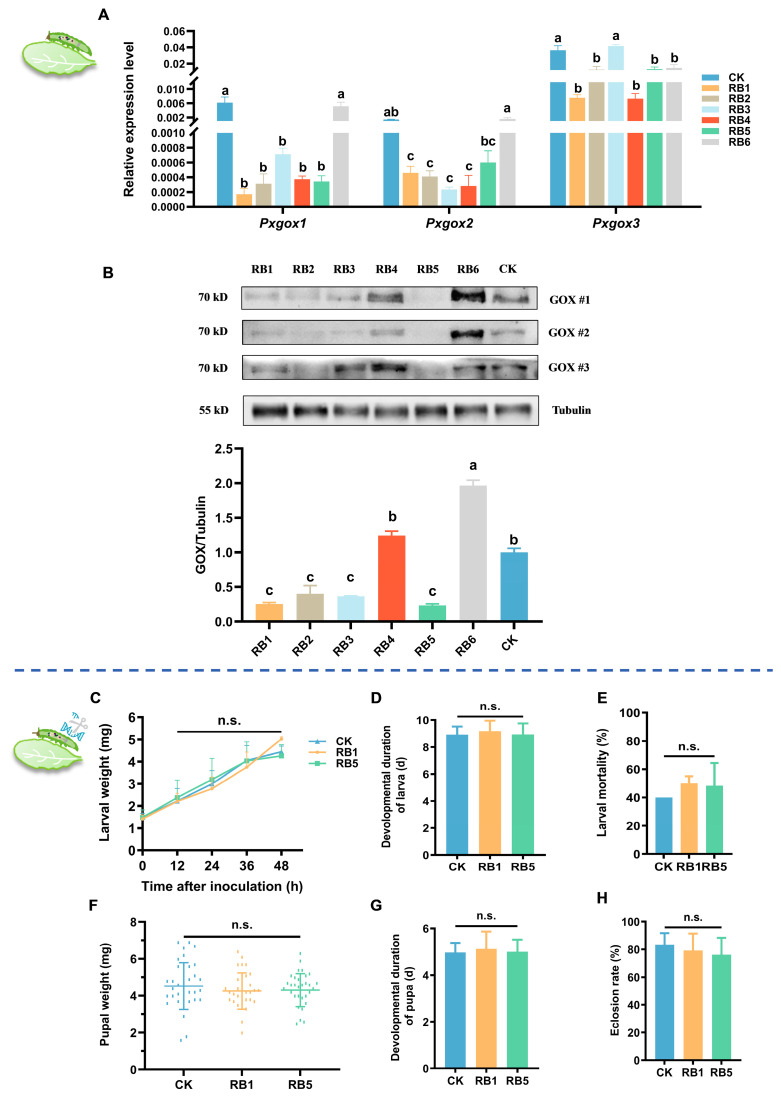
Influence of gut regurgitant bacteria (RB) in GOX-mediated interaction of diamondback moth (DBM) and its host plant. (**A**) Expression profiling of *Pxgoxs* in 3rd instar larvae at 24 hat after different regurgitant bacterium treatments. (**B**) Western blotting analysis of total PxGOXs protein expression in 3rd instar larvae at 24 hat in response to different regurgitant bacterium treatments. The larval weight at 0 h, 12 h, 24 h, 36 h, and 48 h after treatment (**C**), developmental duration of the larva (**D**), larval mortality (**E**), pupal weight (**F**), developmental duration of the pupa (**G**), and eclosion rate (**H**) of the *Pxgox2* mutant with bacteria-free gut and inoculation of RB1, RB5 were compared. CK is larvae with sterile gut without inoculation of bacteria; RB is regurgitant bacteria. Each number of RB represents the larvae inoculated with the corresponding regurgitant bacterium. Due to the high mortality at the larval stage treated by RB2, the biological parameters of the subsequent developmental stages of this treatment were absent. Tubulin was used as standard. Each column represents the mean ± SE (*n* = 3). Different letters indicate significant difference at *p*-value < 0.05. n.s., not significant.

Considering that GOX is the sole effector for functional analysis in DBMs, to further explore the interaction between gut RB and effector proteins in regulating the feeding behavior of DBMs on host plants, we first measured the expression of the *PxGOXs* and GOXs in third instars treated with six different bacterial strains. The expression of *PxGOXs* showed a significant downregulation of *Pxgox1* (*F* = 11.703, *df* = 6, 27, *p* < 0.001), *Pxgox2* (*F* = 8.019, *df* = 6, 27, *p* < 0.001), and *Pxgox3* (*F* = 17.701, *df* = 6, 27, *p* < 0.001) in DBMs treated with RB1, RB2, and RB5 compared to the control group (Figure 7A). Similarly, the expression of GOXs in DBMs treated with RB1, RB2, RB3, and RB5 was significantly inhibited compared to the control group (*F* = 117.824, *df* = 6, 20, *p* < 0.001) (Figure 7B).

Furthermore, in our previous study (Yang et al., 2023), we successfully established the *Pxgox2* mutant strain by CRISPR/Cas9, with a -53bp deletion in the second exon of *Pxgox2*, resulting in a frameshift mutation in the subsequent amino acid sequence (Supplementary Figure S3). Subsequently, we found that the feeding performance (larval weight 0 h (*F*_2, 8_ = 0.074, *df* = 2, 8, *p* = 0.929), 12 h (*F* = 0.078, *df* = 2, 8, *p* = 0.926), 24 h (*F* = 0.249, *df* = 2, 8, *p* = 0.787), 36 h (*F* = 0.156, *df* = 2, 8, *p* = 0.859), 48 h (*F* = 4.447, *df* = 2, 8, *p* = 0.065), developmental duration of larvae (*F* = 0.050, *df* = 2, 29, *p* = 0.952), larval mortality (*F* = 0.912, *df* = 2, 8, *p* = 0.451), pupal weight (*F* = 1.024, *df* = 2, 89, *p* = 0.363), developmental duration of pupa (*F* = 0.528, *df* = 2, 71, *p* = 0.592), and eclosion rate (*F* = 0.325, *df* = 2, 8, *p* = 0.735) of *Pxgox2* mutants inoculated with RB1 and RB5 on *A. thaliana* did not significantly differ from those of bacteria-free individuals (Figure 7C–H).

## 4. Discussion

The larvae of DBMs, as a chewing insect herbivore, secrete gut regurgitant onto plant wounds during feeding. Although many bacteria are speculated to be present in the gut regurgitant, the specific molecular role of RB is unclear. In our study, a total of six culturable bacteria were identified from the gut regurgitant of DBMs, which have also been detected in our previous study on the gut microbiota of DBMs [34]. RB1-3 derived from gut regurgitant are members of the *Enterobacter* genus. *Enterobacter* has been identified from the gut regurgitant of *H. zea*, with the study further revealing that isolates of *Enterobacter asburiae* suppress polyphenol oxidase (PPO) activity in injured tomatoes, *Solanum lycopersicum* [25]. However, *Enterobacter asburiae* (RB3) isolated from the gut regurgitant demonstrates no discernible effect on the performance of DBMs on host plants, suggesting that identical bacteria may exert diverse roles in distinct plant defense reactions. This is analogous to the discovery that *Pantoea*, identified in the secretions of the fall armyworm, *Spodoptera frugiperda*, suppresses the defensive response in tomatoes, but the same bacteria elicits a defensive reaction when the larvae feed on maize [19]. This may imply that insect gut RB is involved in co-evolution between insects and host plants in many ways. Interestingly, we also isolated and identified *Staphylococcus haemolyticus* (RB4) in gut regurgitant, a potentially opportunistic pathogen. *Staphylococcus haemolyticus* was found to act as the potent cellulose-degrading bacteria in the gut of *Brahmina coriacea* grubs [35]. Regarding transboundary associations between insects and bacteria, *Staphylococcus haemolyticus* is a new topic for further research. *Micrococcus luteus* is a Gram-positive bacterium belonging to the phylum Actinobacteria, and this genus has also been identified in the midgut of *H. zea* [36]. In addition, *Micrococcus* was found in the spruce beetle, *Dendroctonus rufipennis*, showing carboxymethylcellulase (CMCase), xylanase and β-glucosidase activities in termites [37,38]. We also isolated and identified *Bacillus cereus* (RB5) from gut regurgitant, a species belonging to the same genus as *Bacillus thuringiensis*, a widely used biological pest control agent, though its potential role as a biocide requires further investigation.

Much evidence suggests that the diversity and abundance of gut bacteria in laboratory-reared insects are commonly lower than in field colonies, possibly attributed to the relatively sterile conditions in laboratory environments and the particular host plant species [36,39]. In addition, adopting artificial feeds has further reduced or altered the diversity of gut bacteria. While this study focused on analyzing the individual effects of six bacterial isolates, testing a mixture of these bacterial isolates would provide valuable insights into potential synergistic or antagonistic interactions among the bacteria. Such a mixed suspension could better simulate the complexity of gut microbiota–host interactions under natural ecological conditions. However, understanding the roles of individual bacteria is a necessary first step for investigating these interactions. Notably, insect gut bacteria may contribute to the interaction between insect immunity and plant immunity by a community of bacteria. In a recent study, *H. zea* larvae inoculated with individual isolated strains did not induce plant anti-herbivore defenses. In contrast, the larvae were inoculated with a bacterial community; there was an increased PPO activity in tomatoes, resulting in delayed larval development [40]. Consequently, future investigations can further explore the relationship between gut microbial communities and their role in modulating interactions between herbivorous insects and their host plants.

To defend against host plants, insects have developed multiple levels of adaptation [7,12,41,42,43]. Among them, the effectors secreted by insects have been the most studied in weakening the plant defense response [44,45,46]. As the chewing insects feed, these secretions enter the plant cells along the wound and play an essential role in interacting with induced plant defense responses [5,11,17]. Bt56 in the tobacco whitefly’s saliva suppresses JA signaling pathways by instigating an antagonistic interplay between the JA and SA signaling pathways [43]. Simultaneously, the effector protein APArmat, secreted into the plant phloem during feeding by the pea aphid, *Acyrthosiphon pisum*, induces an elevation in SA levels, facilitating self-feeding [47,48]. Some studies have found that effectors exist in gut regurgitants in addition to saliva. The nitrile-specific protein (NSP) in the gut of the cabbage white butterfly, *Pieris rapae*, catalyzes the hydrolysis of glucosinolates, leading to nitrile production rather than the toxic secondary substance isothiocyanate generated by myrosinases [49]. Currently, glucosinolate sulfatase 1 (GSS1) and GOX2 are abundantly present in the OS of the DBMs [26,50]. OS is defined as a mixture of all biochemical substances present in insects’ mouthparts and guts [5]. Interestingly, during the feeding process of DBMs, most of the OS residue left at the host plant wound consists of gut regurgitant, with minimal saliva secretion. Therefore, current research on secretions during the feeding process of DBMs mainly focuses on gut regurgitant [18]. A functional analysis indicates that GSS1 can desulfate GSs in vitro, while GOX2 can regulate endogenous plant hormones to help DBMs feed on host plants better [18,44]. JA plays a significant role in the defense against herbivorous insects with chewing mouthparts [25,51,52]. On the other side, SA is involved in the defense response against both living nutritive and semi-living nutritive pathogens [53,54]. The plant defense substance GSs is a potent weapon in cruciferous plants against phytophagous insects feeding on them [55,56,57,58]. Many reports have depicted that the SA and JA defense pathways are antagonistic to each other [59], but some evidence has been presented for a synergistic effect. Therefore, the relationship between these pathways is not clear [51,60]. In general, the local wounding by insect herbivores could cause a rapid increase in JA biosynthesis, which acts as a systemic signal to induce the production of proteinase inhibitors or secondary metabolites with negative effects on pest performance [61,62]. However, the larvae of the colorado potato beetle exploit bacteria in their OS to suppress the JA-dependent defense in the tomato [11]. In our study, when the RB derived from DBM gut were applied on the wounding sites of *A. thaliana*, the expression of JA- and GSs-related genes in *A. thaliana* was also inhibited, implying that gut RB have the potential to directly suppress plant defenses and may influence the defense responses of the host plant by reshaping the phytohormone signaling pathways. Hence, gut RB could interfere with the plant defense response to insect herbivores. However, we observed a significant suppression in the development of larvae treated with RB1 and RB5 while feeding on *A. thaliana*, indicating a difference in the mode of action of gut RB on the insect host and its host plant. Notably, the growth and development of DBMs were not suppressed with RB1 and RB5 treatment while feeding on an artificial diet, suggesting that the host plant could employ these bacteria to suppress the feeding of DBMs. This intricate dynamic implies a sophisticated interplay between the host plant, gut RB, and the herbivorous insects, warranting further investigation into the underlying molecular and physiological mechanisms governing this intriguing ecological interaction.

As widely recognized, bacteria in the gut regurgitant can directly interact with host plants [11,63]. However, insect microbes do not always facilitate insect adaptation to host plants. A prominent example of direct utilization is that *Staphylococcus sciuri* in the pea aphid produces kairomones in the honeydew, attracting natural enemies like the marmalade hoverfly, *Episyrphus balteatus*, thus contributing to protecting the plants [64]. In addition, *Buchnera aphidicola* in the hemocoel of potato aphids, *Macrosiphum euphorbiae*, secretes proteins to the aphid’s saliva. Among these proteins, the chaperonin GroEL is recognized by the plant defense system for triggering an immunity response, which impairs aphid fecundity [65]. Generally, gut bacteria may influence plant defenses by altering the expression of effectors or elicitors in different herbivores. However, it has been revealed that the regurgitant secreted onto plants by fall armyworm larvae does not affect the activity and protein abundance of saliva. It is the bacteria themselves or bacterial-derived components that induce specific plant defense pathways [19]. In the study by Pan et al., inoculating larvae with a mixture of seven isolated bacteria did not impact their growth rate, suggesting no adverse effects on the insects. Moreover, there was no observed effect on the activities of two critical salivary proteins, GOX and phospholipase C [40]. Intriguingly, within the confines of our investigation, a noteworthy discovery was gut RB engaged in indirect interactions with plants. Gut RB indirectly triggered plant defenses by suppressing the expression of GOXs in DBMs, indicating a complicated relationship between bacteria, insects, and plants. The effectors and bacteria are prominently present in the gut regurgitant, secreted into plant wounds as insects feed on the host plants. Investigating the potential interaction between gut RB and effector proteins is of significant importance. To the best of our knowledge, this marks the inaugural study exploring such an interaction in DBMs. Similar to our results, *H. zea* larvae inoculated with *E. ludwigii* induced the expression of JA signaling pathway genes indirectly during feeding on the host plant, enhanced plant resistance to insect defenses, and reduced larval body weight. Interestingly, *E. ludwigii* led to an increase in GOX, thereby inducing an enhanced plant defense response capacity [25,63], which is different from our results that RB1 and RB5 inhibited the development of DBMs by suppressing GOXs. While RB directly suppressed the expression of plant defense pathway genes, it also inhibited the expression of the GOX effector in DBMs. This combined effect tilts the balance in favour of the reduced ability of DBMs to feed on its host plants. This may suggest that gut RB in the insect play a role in the co-evolution between insects and host plants through various mechanisms.

It is of great interest to explore whether interactions exist between gut RB, effectors, and their mechanisms of action. Gut regurgitant may be a significant resource for delivering effector proteins or gut bacteria interacting with host plants. In this study, the RB of the DBM itself inhibited the expression of its effector proteins, implying that gut RB may serve as a “spy” within insect hosts and could be promising candidates for pest control strategies. One limitation of this study is that we exclusively demonstrated the negative regulatory effect of gut RB on GOXs within the gut of DBMs. However, they may exert specific functions when gut RB are released at plant wounds, suppressing the plant’s defense responses. Further investigation into this phenomenon could involve tracking proteins within plants that interact with gut RB, elucidating their functions and mechanisms, which may uncover the intricate and nuanced interactions between plants and gut RB. Undoubtedly, in the direction of insect intestinal microbial pest control, insect pathogenic fungi represent an effective method for controlling pests [66,67]. However, when dealing with pests equipped with chewing mouthparts, the spores nearly lose their ability to germinate after passing through the digestive system of insects, resulting in a meager infection rate [68]. This severely limits their practical application. Utilizing insect gut bacteria for pest control may represent a novel direction. In our study, the culturable bacteria identified from the gut regurgitant of the chewing insect, the DBM, have the potential to indirectly enhance plant resistance by modulating the expression levels of insect effectors. Our findings further reveal a complex and sophisticated tripartite relationship between gut bacteria, insects, and plants, providing insights for understanding ecosystem dynamics between microbes, insects, and plants. Inoculating influential enteric ruminant bacteria into the insect gut to disrupt the insect’s innate immune homeostasis may represent an innovative strategy worthy of intensive research and exploration in future pest biocontrol.

## 5. Conclusions

In summary, we isolated and identified six culturable bacteria from the gut regurgitant of DBMs. All the strains could inhibit the expression of genes associated with the JA and GSs signaling pathways, but only RB1 and RB5 effectively suppressed the expression of the GOXs effectors in DBMs. The *Pxgox2* mutant inoculated with RB1 and RB5 showed no significant changes when DBMs fed on *A. thaliana*, indicating that RB1 and RB5 inhibited the development of DBMs by suppressing GOXs. These findings are expected to propel further investigations, prompting the design and implementation of gut RB-mediated pest management strategies.

## Data Availability

The data presented in this study are available on request from the corresponding author.

## Supplementary Materials

The following supporting information can be downloaded at: https://www.mdpi.com/article/10.3390/insects15121001/s1, Figure S1: Sequence alignment of the six 16S rRNA genes of larval gut regurgitant bacteria (RB) from the diamondback moth (DBM) with the known operational taxonomic unit (OTU) sequences; Figure S2: The adaptive cost of gut regurgitant bacteria (RB) to the artificial diet and *Arabidopsis* of the diamondback moth (DBM); Figure S3: Molecular validation of the *Pxgox2* mutant strain of diamondback moth (DBM); Table S1: Primers used in the molecular characterization; Table S2: Characteristics from the larval regurgitant bacteria (RB) of the diamondback moth; Table S3: Sequence analysis of 16S rRNA from the regurgitant bacteria (RB) of the diamondback moth.
