# Supplementary material for: Bacteria Derived from Diamondback Moth, Plutella xylostella (L.) (Lepidoptera: Plutellidae), Gut Regurgitant Negatively Regulate Glucose Oxidase-Mediated Anti-Defense Against Host Plant
Source: Insects. 2024 Dec 17;15(12):1001. doi: 10.3390/insects15121001 (PMC11677076; doi:10.3390/insects15121001)
Supplement: Supplementary file 1 [file insects-15-01001-s001.zip › Figures S1-3.pdf]

A

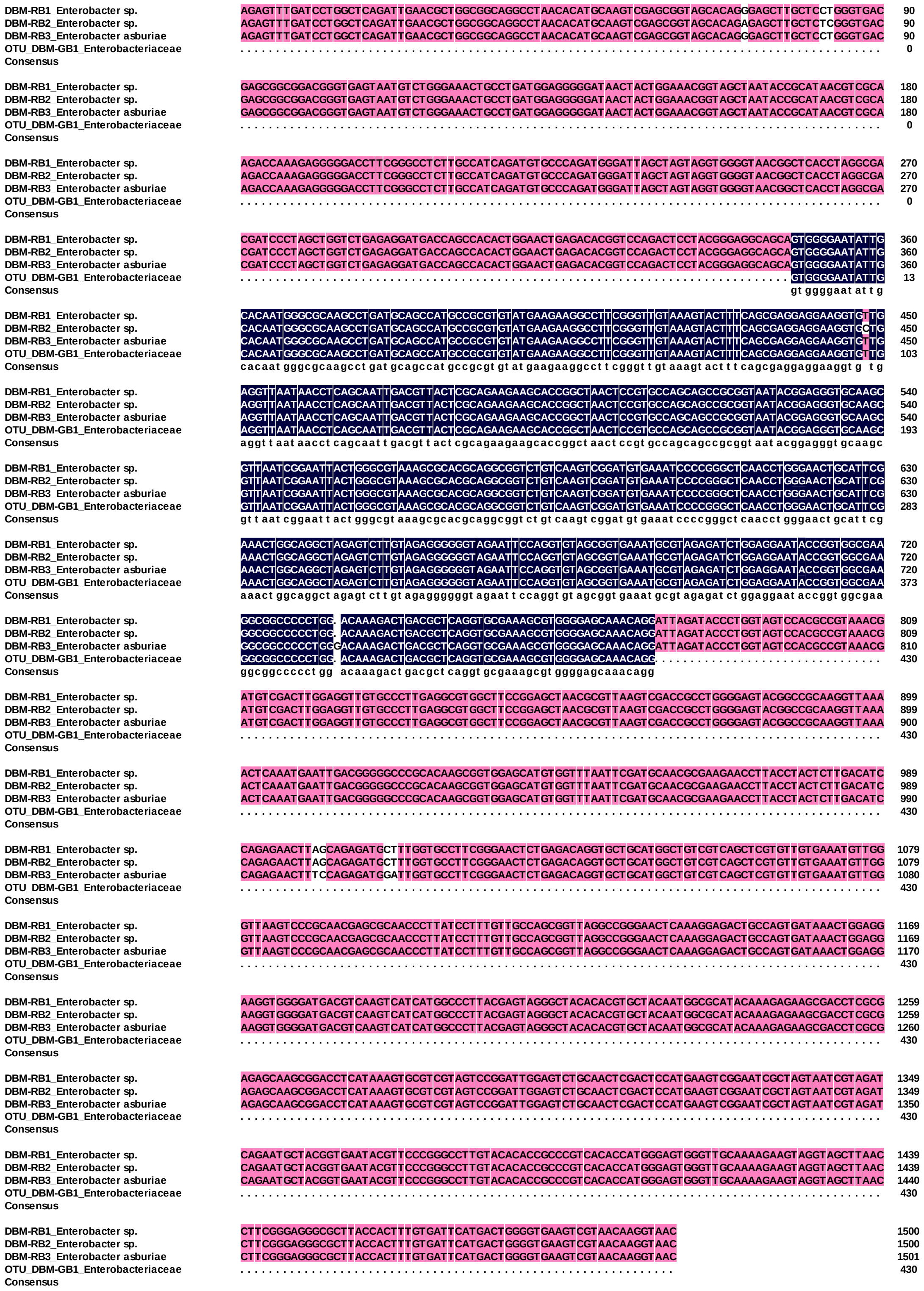

B

```
DBM-RB4_Micrococcus sp.            AGAGTTTGATCCTGGCTCAGGATGAACGCTGGCGGCGTGCTTAACACATGCAAGTCGAACGATGAAGCCCAGCTT     75
OTU_DBM-GB131_Micrococcaceae       ...........................................................................      0
Consensus

DBM-RB4_Micrococcus sp.            GCTGGGTGGATTAGTGGCGAACGGGTGAGTAACACGTGAGTAACCTGCCCTTAACTCTGGGATAAGCCTGGGAAA    150
OTU_DBM-GB131_Micrococcaceae       ...........................................................................      0
Consensus

DBM-RB4_Micrococcus sp.            CTGGGTCTAATACCGGATAGGAGCGCCGACCGCATGGTGGGTGTTGGAAAGATTTATCGGTTTTGGATGGACTCG    225
OTU_DBM-GB131_Micrococcaceae       ...........................................................................      0
Consensus

DBM-RB4_Micrococcus sp.            CGGCCTATCAGCTTGTTGGTGAGGTAATGGCTCACCAAGGCGACGACGGGTAGCCGGCCTGAGAGGGTGACCGGC    300
OTU_DBM-GB131_Micrococcaceae       ...........................................................................      0
Consensus

DBM-RB4_Micrococcus sp.            CACACTGGGACTGAGACACGGCCCAGACTCCTACGGGAGGCAGCAGTGGGGAATATTGCACAATGGGCGCAAGCC    375
OTU_DBM-GB131_Micrococcaceae       .............................................GTGGGGAATATTGCACAATGGGCGCAAGCC     30
Consensus                                                                       gtggggaatattgcacaatgggcgcaagcc

DBM-RB4_Micrococcus sp.            TGATGCAGCGACGCCGCGTGAGGGATGACGGCCTTCGGGTTGTAAACCTCTTTCAGTAGGGAAGAAGCGAAAGTG    450
OTU_DBM-GB131_Micrococcaceae       TGATGCAGCGACGCCGCGTGAGGGATGACGGCCTTCGGGTTGTAAACCTCTTTCAGTAGGGAAGAAGCGAAAGTG    105
Consensus                          tgatgcagcgacgccgcgtgagggatgacggccttcgggttgtaaacctctttcagtagggaagaagcgaaagtg

DBM-RB4_Micrococcus sp.            ACGGTACCTGCAGAAGAAGCACCGGCTAACTACGTGCCAGCAGCCGCGGTAATACGTAGGGTGCGAGCGTTATCC    525
OTU_DBM-GB131_Micrococcaceae       ACGGTACCTGCAGAAGAAGCACCGGCTAACTACGTGCCAGCAGCCGCGGTAATACGTAGGGTGCGAGCGTTATCC    180
Consensus                          acggtacctgcagaagaagcaccggctaactacgtgccagcagccgcggtaatacgtagggtgcgagcgttatcc

DBM-RB4_Micrococcus sp.            GGAATTATTGGGCGTAAAGAGCTCGTAGGCGGTTTGTCGCGTCTGTCGTGAAAGTCCGGGGCTTAACCCCGGATC    600
OTU_DBM-GB131_Micrococcaceae       GGAATTATTGGGCGTAAAGAGCTCGTAGGCGGTTTGTCGCGTCTGCCGTGAAAGTCCGGGGCTTAACTCCGGATC    255
Consensus                          ggaattattgggcgtaaagagctcgtaggcggtttgtcgcgtctg cgtgaaagtccggggcttaac ccggatc

DBM-RB4_Micrococcus sp.            TGCGGTGGGTACGGGCAGACTAGAGTGCAGTAGGGGAGACTGGAATTCCTGGTGTAGCGGTGGAATGCGCAGATA    675
OTU_DBM-GB131_Micrococcaceae       TGCGGTGGGTACGGGCAGACTAGAGTGCAGTAGGGGAGACTGGAATTCCTGGTGTAGCGGTGGAATGCGCAGATA    330
Consensus                          tgcggtgggtacgggcagactagagtgcagtaggggagactggaattcctggtgtagcggtggaatgcgcagata

DBM-RB4_Micrococcus sp.            TCAGGAGGAACACCGATGGCGAAGGCAGGTCTCTGGGCTGTAACTGACGCTGAGGAGCGAAAGCATGGGGAGCGA    750
OTU_DBM-GB131_Micrococcaceae       TCAGGAGGAACACCGATGGCGAAGGCAGGTCTCTGGGCTGTAACTGACGCTGAGGAGCGAAAGCATGGGGAGCGA    405
Consensus                          tcaggaggaacaccgatggcgaaggcaggtctctgggctgtaactgacgctgaggagcgaaagcatggggagcga

DBM-RB4_Micrococcus sp.            ACAGGATTAGATACCCTGGTAGTCCATGCCGTAAACGTTGGGCACTATGTGTGGGGACCATTCCACGGTTTCCGC    825
OTU_DBM-GB131_Micrococcaceae       ACAGG......................................................................    410
Consensus                          acagg

DBM-RB4_Micrococcus sp.            GCCGCAGCTAACGCATTAAGTGCCCCGCCTGGGGAGTACGGCCGCAAGGCTAAAACTCAAAGGAATTGACGGGGG    900
OTU_DBM-GB131_Micrococcaceae       ...........................................................................    410
Consensus

DBM-RB4_Micrococcus sp.            CCCGCACAAGCGGCGGAGCATGCGGATTAATTCGATGCAACGCGAAGAACCTTACCAAGGCTTGACATGTTCTCG    975
OTU_DBM-GB131_Micrococcaceae       ...........................................................................    410
Consensus

DBM-RB4_Micrococcus sp.            ATCGCCGTAGAGATACGGTTTCCCCTTTGGGGCGGGTTCACAGGTGGTGCATGGTTGTCGTCAGCTCGTGTCGTG   1050
OTU_DBM-GB131_Micrococcaceae       ...........................................................................    410
Consensus

DBM-RB4_Micrococcus sp.            AGATGTTGGGTTAAGTCCCGCAACGAGCGCAACCCTCGTTCCATGTTGCCAGCACGTAATGGTGGGGACTCATGG   1125
OTU_DBM-GB131_Micrococcaceae       ...........................................................................    410
Consensus

DBM-RB4_Micrococcus sp.            GAGACTGCCGGGGTCAACTCGGAGGAAGGTGAGGACGACGTCAAATCATCATGCCCCTTATGTCTTGGGCTTCAC   1200
OTU_DBM-GB131_Micrococcaceae       ...........................................................................    410
Consensus

DBM-RB4_Micrococcus sp.            GCATGCTACAATGGCCGGTACAATGGGTTGCGATACTGTGAGGTGGAGCTAATCCCAAAAAGCCGGTCTCAGTTC   1275
OTU_DBM-GB131_Micrococcaceae       ...........................................................................    410
Consensus

DBM-RB4_Micrococcus sp.            GGATTGGGGTCTGCAACTCGACCCCATGAAGTCGGAGTCGCTAGTAATCGCAGATCAGCAACGCTGCGGTGAATA   1350
OTU_DBM-GB131_Micrococcaceae       ...........................................................................    410
Consensus

DBM-RB4_Micrococcus sp.            CGTTCCCGGGCCTTGTACACACCGCCCGTCAAGTCACGAAAGTCGGTAACACCCGAAGCCGGTGGCCTAACCCTT   1425
OTU_DBM-GB131_Micrococcaceae       ...........................................................................    410
Consensus

DBM-RB4_Micrococcus sp.            GTGGGGGGAGCCGTCGAAGGTGGGACCAGCGATTGGGACTAAGTCGTAACAAGGTAAC                    1483
OTU_DBM-GB131_Micrococcaceae       ..........................................................                     410
Consensus
```

C

```
DBM-RB5_Staphylococcus haemolyticus              AGAGTTTGATCCTGGCTCAGGATGAACGCTGGCGGCGTGCCTAATACATGCAAGTCGAGCGAACAGATAAGGAGC  75
OTU_DBM_GB236_Staphylococcus epidermidis         ...........................................................................  0
Consensus

DBM-RB5_Staphylococcus haemolyticus              TTGCTCCTTTGACGTTAGCGGCGGACGGGTGAGTAACACGTGGGTAACCTACCTATAAGACTGGGATAACTTCGG  150
OTU_DBM_GB236_Staphylococcus epidermidis         ...........................................................................  0
Consensus

DBM-RB5_Staphylococcus haemolyticus              GAAACCGGAGCTAATACCGGATAATATTTCGAACCGCATGGTTCGATAGTGAAAGATGGTTTTGCTATCACTTAT  225
OTU_DBM_GB236_Staphylococcus epidermidis         ...........................................................................  0
Consensus

DBM-RB5_Staphylococcus haemolyticus              AGATGGACCCGCGCCGTATTAGCTAGTTGGTAAGGTAACGGCTTACCAAGGCGACGATACGTAGCCGACCTGAGA  300
OTU_DBM_GB236_Staphylococcus epidermidis         ...........................................................................  0
Consensus

DBM-RB5_Staphylococcus haemolyticus              GGGTGATCGGCCACACTGGAACTGAGACACGGTCCAGACTCCTACGGGAGGCAGCAGTAGGGAATCTTCCGCAAT  375
OTU_DBM_GB236_Staphylococcus epidermidis         ........................................................GTAGGGAATCTTCCGCAAT  19
Consensus                                                                                                gtagggaatcttccgcaat

DBM-RB5_Staphylococcus haemolyticus              GGGCGAAAGCCTGACGGAGCAACGCCGCGTGAGTGATGAAGGTCTTCGGATCGTAAAACTCTGTTATTAGGGAAG  450
OTU_DBM_GB236_Staphylococcus epidermidis         GGGCGAAAGCCTGACGGAGCAACGCCGCGTGAGTGATGAAGGTCTTCGGATCGTAAAACTCTGTTATTAGGGAAG  94
Consensus                                        gggcgaaagcctgacggagcaacgccgcgtgagtgatgaaggtcttcggatcgtaaaactctgttattagggaag

DBM-RB5_Staphylococcus haemolyticus              AACATACGTGTAAGTAACTGTGCACGTCTTGACGGTACCTAATCAGAAAGCCACGGCTAACTACGTGCCAGCAGC  525
OTU_DBM_GB236_Staphylococcus epidermidis         AACAAATGTGTAAGTAACTATGCACGTCTTGACGGTACCTAATCAGAAAGCCACGGCTAACTACGTGCCAGCAGC  169
Consensus                                        aaca a gtgtaagtaact tgcacgtcttgacggtacctaatcagaaagccacggctaactacgtgccagcagc

DBM-RB5_Staphylococcus haemolyticus              CGCGGTAATACGTAGGTGGCAAGCGTTATCCGGAATTATTGGGCGTAAAGCGCGCGTAGGCGGTTTTTTAAGTCT  600
OTU_DBM_GB236_Staphylococcus epidermidis         CGCGGTAATACGTAGGTGGCAAGCGTTATCCGGAATTATTGGGCGTAAAGCGCGCGTAGGCGGTTTTTTAAGTCT  244
Consensus                                        cgcggtaatacgtaggtggcaagcgttatccggaattattgggcgtaaagcgcgcgtaggcggttttttaagtct

DBM-RB5_Staphylococcus haemolyticus              GATGTGAAAGCCCACGGCTCAACCGTGGAGGGTCATTGGAAACTGGAAAACTTGAGTGCAGAAGAGGAAAGTGGA  675
OTU_DBM_GB236_Staphylococcus epidermidis         GATGTGAAAGCCCACGGCTCAACCGTGGAGGGTCATTGGAAACTGGAAAACTTGAGTGCAGAAGAGGAAAGTGGA  319
Consensus                                        gatgtgaaagcccacggctcaaccgtggagggtcattggaaactggaaaacttgagtgcagaagaggaaagtgga

DBM-RB5_Staphylococcus haemolyticus              ATTCCATGTGTAGCGGTGAAATGCGCAGAGATATGGAGGAACACCAGTGGCGAAGGCGACTTTCTGGTCTGTAAC  750
OTU_DBM_GB236_Staphylococcus epidermidis         ATTCCATGTGTAGCGGTGAAATGCGCAGAGATATGGAGGAACACCAGTGGCGAAGGCGACTTTCTGGTCTGTAAC  394
Consensus                                        attccatgtgtagcggtgaaatgcgcagagatatggaggaacaccagtggcgaaggcgactttctggtctgtaac

DBM-RB5_Staphylococcus haemolyticus              TGACGCTGATGTGCGAAAGCGTGGGGATCAAACAGGATTAGATACCCTGGTAGTCCACGCCGTAAACGATGAGTG  825
OTU_DBM_GB236_Staphylococcus epidermidis         TGACGCTGATGTGCGAAAGCGTGGGGATCAAACAGG.......................................  430
Consensus                                        tgacgctgatgtgcgaaagcgtggggatcaaacagg

DBM-RB5_Staphylococcus haemolyticus              CTAAGTGTTAGGGGGTTTCCGCCCCTTAGTGCTGCAGCTAACGCATTAAGCACTCCGCCTGGGGAGTACGACCGC  900
OTU_DBM_GB236_Staphylococcus epidermidis         ...........................................................................  430
Consensus

DBM-RB5_Staphylococcus haemolyticus              AAGGTTGAAACTCAAAGGAATTGACGGGGACCCGCACAAGCGGTGGAGCATGTGGTTTAATTCGAAGCAACGCGA  975
OTU_DBM_GB236_Staphylococcus epidermidis         ...........................................................................  430
Consensus

DBM-RB5_Staphylococcus haemolyticus              AGAACCTTACCAAATCTTGACATCCTTTGACAACTCTAGAGATAGAGTTTTCCCCTTCGGGGGACAAAGTGACAG  1050
OTU_DBM_GB236_Staphylococcus epidermidis         ...........................................................................  430
Consensus

DBM-RB5_Staphylococcus haemolyticus              GTGGTGCATGGTTGTCGTCAGCTCGTGTCGTGAGATGTTGGGTTAAGTCCCGCAACGAGCGCAACCCTTAAGCTT  1125
OTU_DBM_GB236_Staphylococcus epidermidis         ...........................................................................  430
Consensus

DBM-RB5_Staphylococcus haemolyticus              AGTTGCCATCATTAAGTTGGGCACTCTAAGTTGACTGCCGGTGACAAACCGGAGGAAGGTGGGGATGACGTCAAA  1200
OTU_DBM_GB236_Staphylococcus epidermidis         ...........................................................................  430
Consensus

DBM-RB5_Staphylococcus haemolyticus              TCATCATGCCCCTTATGATTTGGGCTACACACGTGCTACAATGGACAATACAAAGGGTAGCGAAACCGCGAGGTC  1275
OTU_DBM_GB236_Staphylococcus epidermidis         ...........................................................................  430
Consensus

DBM-RB5_Staphylococcus haemolyticus              AAGCAAATCCCATAAAGTTGTTCTCAGTTCGGATTGTAGTCTGCAACTCGACTACATGAAGCTGGAATCGCTAGT  1350
OTU_DBM_GB236_Staphylococcus epidermidis         ...........................................................................  430
Consensus

DBM-RB5_Staphylococcus haemolyticus              AATCGTAGATCAGCATGCTACGGTGAATACGTTCCCGGGTCTTGTACACACCGCCCGTCACACCACGAGAGTTTG  1425
OTU_DBM_GB236_Staphylococcus epidermidis         ...........................................................................  430
Consensus

DBM-RB5_Staphylococcus haemolyticus              TAACACCCGAAGCCGGTGGAGTAACCATTTGGAGCTAGCCGTCGAAGGTGGGACAAATGATTGGGGTGAAGTCGT  1500
OTU_DBM_GB236_Staphylococcus epidermidis         ...........................................................................  430
Consensus

DBM-RB5_Staphylococcus haemolyticus              AACAAGGTAAC                                                                  1511
OTU_DBM_GB236_Staphylococcus epidermidis         ...........                                                                  430
Consensus
```

D

```
DBM-RB6_Bacillus cereus     TGCAGTCGAGCGAATGGATTAAGAGCTTGCTCTTATGAAGTTAGCGGCGGACGGGTGAGTAACACGTGGGTAACC     75
OTU_DBM-GB9_Bacillus        ...........................................................................      0
Consensus

DBM-RB6_Bacillus cereus     TGCCCATAAGACTGGGATAACTCCGGGAAACCGGGGCTAATACCGGATAACATTTTGAACCGCATGGTTCGAAAT    150
OTU_DBM-GB9_Bacillus        ...........................................................................      0
Consensus

DBM-RB6_Bacillus cereus     TGAAAGGCGGCTTCGGCTGTCACTTATGGATGGACCCGCGTCGCATTAGCTAGTTGGTGAGGTAACGGCTCACCA    225
OTU_DBM-GB9_Bacillus        ...........................................................................      0
Consensus

DBM-RB6_Bacillus cereus     AGGCAACGATGCGTAGCCGACCTGAGAGGGTGATCGGCCACACTGGGACTGAGACACGGCCCAGACTCCTACGGG    300
OTU_DBM-GB9_Bacillus        ...........................................................................      0
Consensus

DBM-RB6_Bacillus cereus     AGGCAGCAGTAGGGAATCTTCCGCAATGGACGAAAGTCTGACGGAGCAACGCCGCGTGAGTGATGAAGGCTTTCG    375
OTU_DBM-GB9_Bacillus        ........GTAGGGAATCTTCCGCAATGGACGAAAGTCTGACGGAGCAACGCCGCGTGAGTGATGAAGGCTTTCG     67
Consensus                           gt agggaat ct t ccgcaat ggacgaaagt ct gacggagcaacgccgcgt gagt gat gaaggct t t cg

DBM-RB6_Bacillus cereus     GGTCGTAAAACTCTGTTGTTAGGGAAGAACAAGTGCTAGTTGAATAAGCTGGCACCTTGACGGTACCTAACCAGA    450
OTU_DBM-GB9_Bacillus        GGTCGTAAAACTCTGTTGTTAGGGAAGAACAAGTGCTAGTTGAATAAGCTGGCACCTTGACGGTACCTAACCAGA    142
Consensus                   ggt cgt aaaact ct gt t gt t agggaagaacaagt gct agt t gaat aagct ggcacct t gacggt acct aaccaga

DBM-RB6_Bacillus cereus     AAGCCACGGCTAACTACGTGCCAGCAGCCGCGGTAATACGTAGGTGGCAAGCGTTATCCGGAATTATTGGGCGTA    525
OTU_DBM-GB9_Bacillus        AAGCCACGGCTAACTACGTGCCAGCAGCCGCGGTAATACGTAGGTGGCAAGCGTTATCCGGAATTATTGGGCGTA    217
Consensus                   aagccacggct aact acgt gccagcagccgcggt aat acgt aggt ggcaagcgt t at ccggaat t at t gggcgt a

DBM-RB6_Bacillus cereus     AAGCGCGCGCAGGTGGTTTCTTAAGTCTGATGTGAAAGCCCACGGCTCAACCGTGGAGGGTCATTGGAAACTGGG    600
OTU_DBM-GB9_Bacillus        AAGCGCGCGCAGGTGGTTTCTTAAGTCTGATGTGAAAGCCCACGGCTCAACCGTGGAGGGTCATTGGAAACTGGG    292
Consensus                   aagcgcgcgcaggt ggt t t ct t aagt ct gat gt gaaagcccacggct caaccgt ggagggt cat t ggaaact ggg

DBM-RB6_Bacillus cereus     AGACTTGAGTGCAGAAGAGGAAAGTGGAATTCCATGTGTAGCGGTGAAATGCGTAGAGATATGGAGGAACACCAG    675
OTU_DBM-GB9_Bacillus        AGACTTGAGTGCAGAAGAGGAAAGTGGAATTCCATGTGTAGCGGTGAAATGCGTAGAGATATGGAGGAACACCAG    367
Consensus                   agact t gagt gcagaagaggaaagt ggaat t ccat gt gt agcggt gaaat gcgt agagat at ggaggaacaccag

DBM-RB6_Bacillus cereus     TGGCGAAGGCGACTTTCTGGTCTGTAACTGACACTGAGGCGCGAAAGCGTGGGGAGCAAACAGGATTAGATACCC    750
OTU_DBM-GB9_Bacillus        TGGCGAAGGCGACTTTCTGGTCTGTAACTGACACTGAGGCGCGAAAGCGTGGGGAGCAAACAGG...........    431
Consensus                   t ggcgaaggcgact t t ct ggt ct gt aact gacact gaggcgcgaaagcgt ggggagcaaacagg

DBM-RB6_Bacillus cereus     TGGTAGTCCACGCCGTAAACGATGAGTGCTAAGTGTTAGAGGGTTTCCGCCCTTTAGTGCTGAAGTTAACGCATT    825
OTU_DBM-GB9_Bacillus        ...........................................................................    431
Consensus

DBM-RB6_Bacillus cereus     AAGCACTCCGCCTGGGGAGTACGGCCGCAAGGCTGAAACTCAAAGGAATTGACGGGGGCCCGCACAAGCGGTGGA    900
OTU_DBM-GB9_Bacillus        ...........................................................................    431
Consensus

DBM-RB6_Bacillus cereus     GCATGTGGTTTAATTCGAAGCAACGCGAAGAACCTTACCAGGTCTTGACATCCTCTGAAAACCCTAGAGATAGGG    975
OTU_DBM-GB9_Bacillus        ...........................................................................    431
Consensus

DBM-RB6_Bacillus cereus     CTTCTCCTTCGGGAGCAGAGTGACAGGTGGTGCATGGTTGTCGTCAGCTCGTGTCGTGAGATGTTGGGTTAAGTC   1050
OTU_DBM-GB9_Bacillus        ...........................................................................    431
Consensus

DBM-RB6_Bacillus cereus     CCGCAACGAGCGCAACCCTTGATCTTAGTTGCCATCATTAAGTTGGGCACTCTAAGGTGACTGCCGGTGACAAAC   1125
OTU_DBM-GB9_Bacillus        ...........................................................................    431
Consensus

DBM-RB6_Bacillus cereus     CGGAGGAAGGTGGGGATGACGTCAAATCATCATGCCCCTTATGACCTGGGCTACACACGTGCTACAATGGACGGT   1200
OTU_DBM-GB9_Bacillus        ...........................................................................    431
Consensus

DBM-RB6_Bacillus cereus     ACAAAGAGCTGCAAGACCGCGAGGTGGAGCTAATCTCATAAAACCGTTCTCAGTTCGGATTGTAGGCTGCAACTC   1275
OTU_DBM-GB9_Bacillus        ...........................................................................    431
Consensus

DBM-RB6_Bacillus cereus     GCCTACATGAAGCTGGAATCGCTAGTAATCGCGGATCAGCATGCCGCGGTGAATACGTTCCCGGGCCTTGTACAC   1350
OTU_DBM-GB9_Bacillus        ...........................................................................    431
Consensus

DBM-RB6_Bacillus cereus     ACCGCCCGTCACACCACGAGAGTTTGTAACACCCGAAGTCGGTGGGGTAAC                           1401
OTU_DBM-GB9_Bacillus        ...................................................                            431
Consensus
```

Figure S1. Sequence alignment of the six 16S rRNA genes of larval gut regurgitant bacteria (RB) from diamondback moth (DBM) with the known operational taxonomic unit (OTU) sequences

(A) RB1, RB2 and RB3, **(B)** RB4, **(C)** RB5 and **(D)** RB6. RB, regurgitant bacteria. Each number of RB represents the larvae inoculated with the corresponding regurgitant bacterium. The OTU

sequences have been deposited in NCBI under the accession numbers of SRR11206458–SRR11206466.

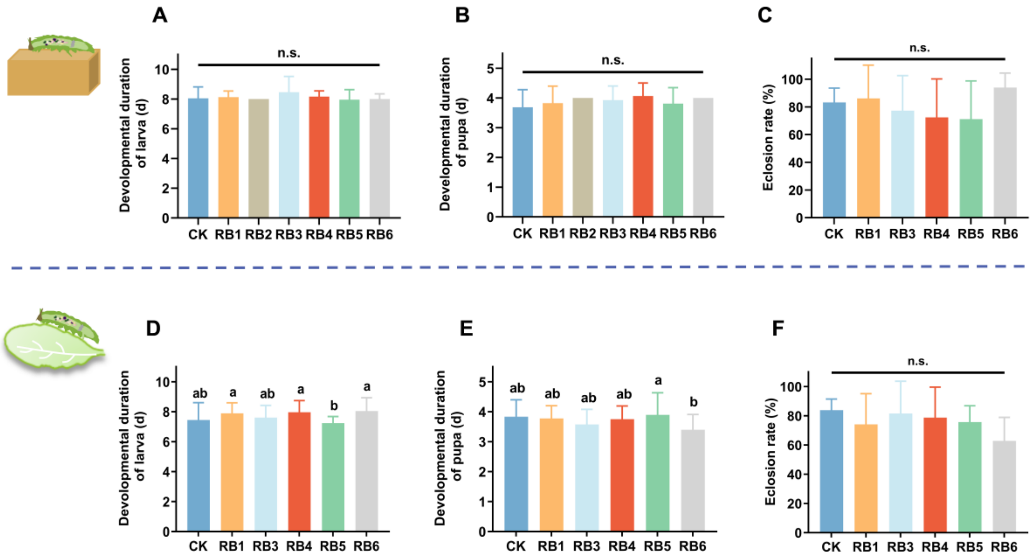

Figure S2. The adaptive cost of gut regurgitant bacteria (RB) to the artificial diet and *Arabidopsis* of diamondback moth (DBM)

The developmental duration of larva **(A and D)**, developmental duration of pupa **(B and E)**, and eclosion rate **(C and F)** were compared for the larvae reared on artificial diet and host plant *Arabidopsis*, each of which has been inoculated with different single strain of the bacteria isolated from larval regurgitant of DBM. CK, the larvae with sterile gut without inoculation of bacteria; RB, regurgitant bacteria. Each number of RB represents the larvae inoculated with the corresponding regurgitant bacterium. Due to the high mortality at larval stage treated by RB2, the biological parameters of subsequent developmental stages of this treatment were absent. Each column represents mean ± SE (n=3). Significant difference analysis for comparison between CK and each treatment group was performed using one-way ANOVA followed by Tukey's post-hoc test, which was taken to be significant at a *P-value* < 0.05. n.s., not significant.

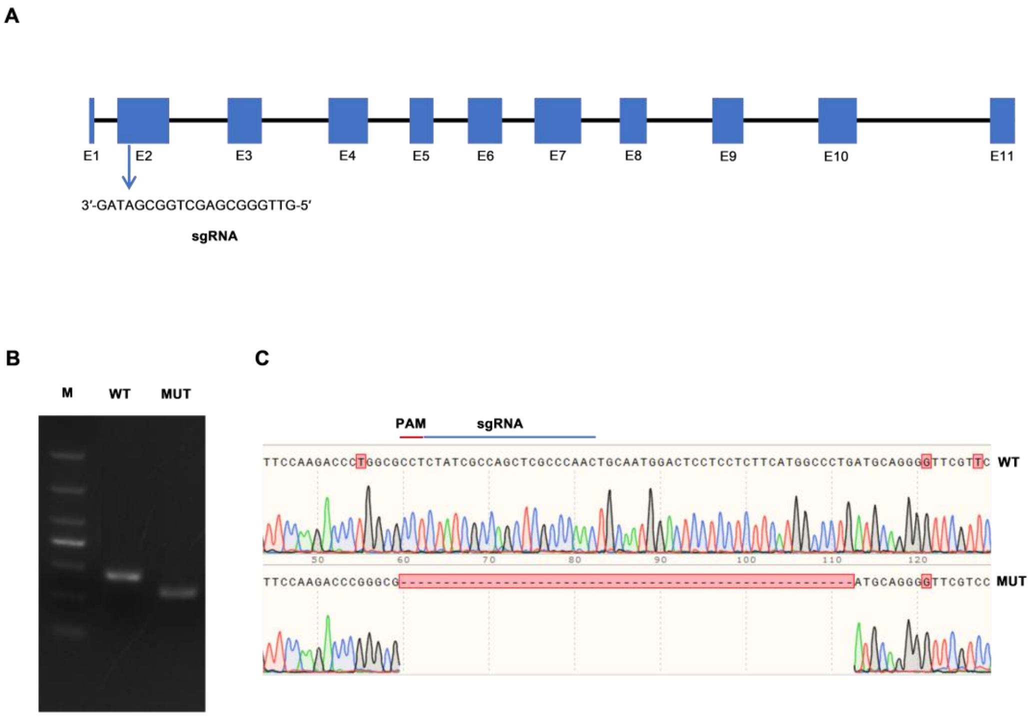

Figure S3. Molecular validation of the *Pxgox2* mutant strain of diamondback moth (DBM)

(A) Schematic presentation of the location of sgRNA targeting the 2nd exon of *Pxgox2*. **(B)** Detection of mutation in *Pxgox2* based on by agarose gel electrophoresis at the DNA level. WT, wild-type strain of DBM reared on artificial diet; MUT, -53bp deletion mutation of *Pxgox2* of DBM and reared on artificial diet. **(C)** Validation of mutation in *Pxgox2* based on Sanger sequencing.
