# Supplementary material for: Bacteria Derived from Diamondback Moth, Plutella xylostella (L.) (Lepidoptera: Plutellidae), Gut Regurgitant Negatively Regulate Glucose Oxidase-Mediated Anti-Defense Against Host Plant
Source: Insects. 2024 Dec 17;15(12):1001. doi: 10.3390/insects15121001 (PMC11677076; doi:10.3390/insects15121001)
Supplement: Supplementary file 1 [file insects-15-01001-s001.zip › Tables S1-3.pdf]

Table S1 Primers used in the molecular characterization

Primer[1]	Sequence (5′ – 3′)	Description
27F	AGAGTTTGATCCTGGCTCAG	Bacterial 16S rRNA gene
1492R	GGTTACCTTGTTACGACTT	
PxRibl32-F	CAATCAGGCCAATTTACCGC	Reference for qRT-PCR of *P. xylostella*
PxRibl32-R	CTGGGTTTACGCCAGTTACG	
AtAoc1-F	ACGGTCCAGGTTCCTCCTCT	qRT-PCR of genes involved in JA biosynthesis and signaling
AtAOC1-R	ACTTTGCTTGGTCTGGGGTTT	
AtLox2-F	ACCAACGACAACAAGGATAAGAATG	
AtLox2-R	CCTGTTTCTGCGATGGGTATG	
AtAos-F	GATTCGTCGGAGAAGAAGGAGA	
AtAos-R	CTCGCCACCAAAACAACAAA	
AtOpr3-F	TTCGGTCCAAGATGCCAAG	
AtOpr3-R	TGTGTTGTTGTTGTGTGGTCGT	
AtJar1-F	GAAACGCTACTGACCCTGAAGAA	
AtJar1-R	AAGGAAATGGCAGGAACAGG	
AtEds5-F	TTGGGAGTTATCGGAACAGC	qRT-PCR of genes involved in SA signaling and response
AtEds5-R	GACAACGCCATGAAGAAAGG	
AtPad4-F	AGATACGCGAGCACAACGCAAG	
AtPad4-R	TTCTCGCCTCATCCAACCACTC	
AtSid2-F	GCGAGGAGAGTGAATTTGCAGTCG	
AtSid2-R	CCACTCTGAAGATGGGTCACTTCCA	
AtPr1-F	TTACTGGCTATTCTCGATTTTTAATC	
AtPr1-R	TATCTTGAGCTTTCGAGGGAA	
AtPr5-F	TTGCTGTTATGGCCACAGACTT	
AtPr5-R	TCCTTGACCGGCGAGAGTT	
AtBgl2-F	CACTGACACCACCACTGATA	
AtBgl2-R	ATTCACGAGCAAGGGAGATT	

Table S1 Primers used in the molecular characterization

Primer	Sequence (5′ – 3′)	Description
AtCyp79a2-F	GGAAAAGACCGTCTTGTCAT	qRT-PCR of gene involved in biosynthesis of aromatic glucosinolates
AtCyp79a2-R	GGTGTAACCGGAATGCTTCT	
Atcyp79b2-F	AGGAAAGAGAACTCAAATCG	
Atcyp79b2-R	ATCTCTTCCATTGCTTTACG	
Atcyp79b3-F	CGCCAGACAACCCATCAA	
Atcyp79b3-R	GACGGGATGAAGACGGAAAG	
Atmyb29-F	TCATAGCGAGACATTTGCC	
Atmyb29-R	TCCCTTATCGATCAGGAGC	
Atmyb28-F	CCATGTTGCGTCGGAGAA	
Atmyb28-R	GTGGTCGTGGATGTAAGAGATGAG	
Attgg1-F	ATATTTTTCCATGGATGGTTCATG	
AtTgg1-R	CAAGTGCGGCTTCTGTTTCAC	
AtActin2-F	AGGAGATGGAAACCTCAAAGACC	Reference for qRT-PCR of *A. thaliana*
AtActin2-R	AAACGAGGGCTGGAACAAGA	
gox2-sgRNA	*TAATACGACTCACTATAGG*GAAGGCTCCGATCTGAGACG<u>GTTTTAGAGCTAGAAATAGCAAGTTAAAATAAGGCTAGTCC</u>	SgRNA synthesis[2]
*gox*2-common R	AAAAGCACCGACTCGGTGCCACTTTTTCAAGTTGATAACGGACTAGCCTTATTTTAACTTGCTATTTCTAGCTCTAAAA	
test-*gox2*-F	TGATTCTAGCTCCAGAGGTATG	Mutant detection
test-*gox2*-R	TGATTCTAGCTCCAGAGGTATG	

[1]The prefix of *Px* denotes the sequences of *Plutella xylostella*, and *At* represents *Arabidopsis thaliana*.

[2]The T7 polymerase-binding site was in italic. SgRNA is represented by wavy lines. The sequences complementary to sgRNA scaffold were underlined.

Table S2 Characteristics from the larval regurgitant bacteria (RB) of diamondback moth

Numbers	Cultural features
RB1	The colony morphology was round, white, translucent, smooth, and moist, with irregular and transparent edges.
RB2	The colony morphology was round and relatively large, white, translucent, smooth, and moist, with irregular and slightly transparent edges.
RB3	The colony morphology was round, rice white, translucent, smooth, and moist, with neat and opaque edges.
RB4	The colony morphology was round, light yellow, and opaque, with a smooth surface and neat edge.
RB5	The colony morphology was round, grayish white, opaque, smooth surface, and neat edge.
RB6	The colony morphology was round, grayish-white, and opaque; the surface was wet, and the edge was not neat.

Table S3 Sequences analysis of 16S rRNA from the regurgitant bacteria (RB) of diamondback moth

Isolate strains	Genbank No.	Closest related species	Identity (%)
RB1	MT321481	*Enterobacter* sp1. (KT957443.1)	99.80
RB2	MT321505	*Enterobacter* sp2. (KT957443.1)	99.73
RB3	MT321506	*Enterobacter asburiae* (CP046618.1)	99.93
RB4	MT321507	*Micrococcus* sp. (AB188213.1)	99.93
RB5	MT321508	*Staphylococcus haemolyticus* (KT696497.1)	99.93
RB6	MT321509	*Bacillus cereus* (MT214211.1)	100.00
